# Religious psychopathology: The prevalence of religious content of delusions and hallucinations in mental disorder

**DOI:** 10.1177/0020764015573089

**Published:** 2015-06

**Authors:** Christopher CH Cook

**Affiliations:** Department of Theology and Religion, Durham University, Durham, UK

**Keywords:** Spirituality, religion, delusions, hallucinations

## Abstract

**Background:**

Religious themes are commonly encountered in delusions and hallucinations associated with major mental disorders, and the form and content of presentation are significant in relation to both diagnosis and management.

**Aims:**

This study aimed to establish what is known about the frequency of occurrence of religious delusions (RD) and religious hallucinations (RH) and their inter-relationship.

**Methods:**

A review was undertaken of the quantitative empirical English literature on RD and RH.

**Results:**

A total of 55 relevant publications were identified. The lack of critical criteria for defining and classifying RD and RH makes comparisons between studies difficult, but prevalence clearly varies with time and place, and probably also according to personal religiosity. In particular, little is known about the content and frequency of RH and the relationship between RH and RD.

**Conclusion:**

Clearer research criteria are needed to facilitate future study of RD and RH, and more research is needed on the relationship between RD and RH.

## Introduction

As a branch of medicine, psychiatry is concerned not only with trying to understand mental disorders but also with trying to find treatments to alleviate the suffering and stigma with which they are so notoriously associated. This concern with treatment underlies a concern for diagnosis, as it is through arriving at a diagnosis that prognosis can be predicted and the most appropriate treatment selected in any given case. Diagnosis in psychiatry is primarily based upon information gained from the history and from the mental state examination, both of which require a degree of trust between doctor and patient, and a sensitivity of the clinician to diagnostic clues which must be interpreted according to the culture and context in which the patient lives. An important component of this culture and context, even in a secular society, is contributed by religious tradition. Unfortunately, the relationship between psychiatry and religion has at times been fraught, and patients have not always felt that they could entrust their psychiatrist with a frank account of their religious experiences, for fear that such experiences might be used as evidence to make a diagnosis of mental illness. The situation has not been helped by crude attempts to employ psychiatric concepts for diagnosing saints and mystics as mentally ill (Allen, 1975; Cook, 2012).

In major mental disorder, the content of perceptual disorder and thought disorder has often assumed less diagnostic significance than the form of the disorder. Thus, it is the presence of a false perception that is understood as important, rather than whether the content of the perception is religious, political or scientific. Similarly, it is the falseness of unshakeable beliefs which are out of keeping with culture that renders them delusional, rather than that they are religious (or political or of another kind). This might be thought to assist in preventing normal religious or political beliefs from being used as a basis for diagnosis. However, it can also lead to a lack of interest of the clinician in religious or other significant themes which may be of central importance to the patient. This is despite evidence that religion may provide an important coping resource for people suffering from major mental disorder (Mohr et al., 2010) and may significantly influence adherence to treatment (Borras et al., 2007).

Studies have generally found religious themes to be commonly identifiable within the content of delusional beliefs, and some helpful reviews have been published (Bhavsar & Bhugra, 2008; Gearing et al., 2011). Religious delusions (RD) may be associated with higher levels of grandiosity, but are also held with a degree of flexibility that may give reason to believe that they may be more amenable to cognitive behaviour therapy (Iyassu et al., 2014). Delusion-like beliefs, including some with religious content, are held widely in the general population, and so RD might be considered as one end of a spectrum of belief, with ‘normal’ religious beliefs at the opposite end of the spectrum (Pechey & Halligan, 2011). It has been suggested that RD are becoming less common in the Western world as religion has declined in popularity (Stompe, Ortwein-Swoboda, Ritter, & Schanda, 2003). Widely varying figures have been quoted for the prevalence of RD, and few attempts appear to have been made to systematically review this literature (none of which have attempted to be comprehensive). A large number of such studies have now been published.

Much less attention has been given to the religious content of hallucinations, and little is known about the frequency of occurrence of religious content as a feature of such phenomena. However, at least one attempt has been made to conduct a systematic and comprehensive review (Gearing et al., 2011). Some attention has been given to the phenomenon of voice hearing occurring in the absence of diagnosable mental illness, including the occurrence of such phenomena in religious populations. In such a context, it appears that healthy individuals do report, at least sometimes, hearing the voice of God (Dein & Littlewood, 2007; Luhrmann, 2012b). Little is known about the frequency of occurrence of religious themes in hallucinations occurring in the course of mental disorder.

This study sought to review the empirical literature pertaining to the frequency of religious content of hallucinations and delusions as a feature of mental disorders.

## Methodology

Attempts were made to ascertain relevant studies by searching bibliographic databases such as MEDLINE and PsycINFO. This was not found to be a helpful approach as large numbers of studies already known to the author were not identified by this means and it was difficult to identify any search terms which located other than very small numbers of relevant empirical studies. Accordingly, reliance was placed initially upon known review papers which referenced relevant articles on RD and/or religious hallucinations (RH). Further studies were identified by a variety of means, notably by following up references from journal articles and book chapters already identified, by careful attention to recent publications in the field and by searching the MEDLINE and PsycINFO databases with a variety of different free text terms. While it is impossible to be sure that all relevant studies have been identified, the active search for older publications was discontinued when no new articles were being located despite extensive efforts to search manually and by using available electronic databases.

Inclusion criteria for the articles that were identified included primarily that they were empirical studies which included at least some data on frequency of religious content of delusions and/or hallucinations in the population studied. Individual case reports, and case reports of very small numbers of subjects (*n* < 10), were not included. The study was restricted to articles published in English (with the exception of one paper in Korean, with results tables published in English). Qualitative and quantitative studies were included, but only where data allowed at least a basic quantitative calculation of the number of subjects with religious psychopathology. The primary focus was on studies providing data on RH and RD. Studies on religious rituals and obsessional ruminations, other anxiety disorders, non-psychotic affective disorder, eating disorders and religious addiction were not included.

## Results

A total of 55 publications were identified as meeting inclusion criteria and were included in the study (see Table 1). Of these, 45 publications provided at least some quantitative information on numbers of subjects with RD (see Table 2) and 28 provided at least some information (qualitative or quantitative) on the occurrence and nature of RH (see Table 2). The two publications by Kala and Wig (1978, 1982), appearing in Tables 1 and 2, would appear to relate to the same study – although slightly different results are published in each paper.

**Table 1.** Overview of empirical studies of religious delusions and hallucinations.

Publication	Country	Study subjects			Age		Ethnicity	Religion	Diagnosis	Ascertainment	Methodology	RD	Hallucinations
		Total, *n*	Male, *n*	Female, *n*	Range	Mean							
Lucas et al. (1962)	England	405	196	209	NK	49.5 (M) 53.3 (F)			S, S-aff, Par	ip in 1st half of 1958	IS	✓	
Rin et al. (1962)	Taiwan – Chinese	126	52	74	NK	NK	✓		Par	ip, 1948–1959	CR	✓	[✓]
	Taiwan – Formosan	94	45	49	NK	NK							
Weinstein (1962)	Virgin Islands	148	83	65	NK	NK	✓		P	ip	IS	✓	[✓]
Kiev (1963)	England	10	NK	NK	25–35	NK	✓	[✓]	S	ip at least 1 year	IS	✓	[✓]
Gordon (1965)	England	112	61	51	>15	NK	✓		S, S-form, Aff, S-aff, Org, PD, N	ip, 1961–1964	IS and CR	✓	[✓]
Mott, Small, and Anderson (1965)	United States	50	14	36	NK	33.2			S	ip	IS		✓
		50	44	6	NK	42.4			SA (alcoholic)				
		50	26	24	NK	56.0			Medical				
Scott (1967)	South Africa	100	0	100	NK	NK	✓		P	2nd week of January 1966	IS	[✓]	✓
McCabe, Fowler, Cadoret, and Winokur (1972)	United States	28	8	20	16–77	31.4	✓		Good prognosis S	Consecutive ip	IS	✓	✓
		25	10	15	17–55	32.3			Poor prognosis S				
El Sendiony (1976)	Egypt	110	56	54	NK	NK		[✓]	S, Par	ip	CR	✓	
Ahmed (1978)	Pakistan	51	31	20	16–55	30		[✓]	S	op	IS	✓	
Kala and Wig (1978)	India	200	107	93	NK	NK			ICD8: S, Par	Consecutive op, 10 January–15 September 1974	IS	✓	
Littlewood and Lipsedge (1981)	England	244	NK	NK	15–45	NK	✓		S, Aff, Par, Other P, PD	Consecutive ip	CR	[✓]	[✓]
Kala and Wig (1982)	India	200	109	93	NK	NK			ICD8: S, Par	Consecutive op	IS	✓	
Ndetei and Singh (1982)	Kenya	80	NK	NK	15–65	27.7	✓		All psych	ip	IS	✓	
Ndetei and Vadher (1985)	England	593	NK	NK	NK	NK	✓	✓	All psych	ip	CR	✓	
Cothran and Harvey (1986)	United States	41	NK	NK	NK	NK		✓	DSMIII: S, Mania	Consecutive ip	IS	✓	
Kulhara et al. (1986)	India	112	59	53	>14	27.7			ICD9: S	NK	IS	✓	
Andreasen (1987)	United States	111	NK	NK	NK	NK			S	Consecutive ip	IS	✓	[✓]
Mitchell and Vierkant (1988)	United States	150	89	61	>16	NK	✓	[✓]	NK	ip, 1933–1939	CR	[✓]	✓
		150	89	61						ip, 1986 and 1987			
Renvoize and Beveridge (1989)	England	118	54	64	20–79	NK		✓	All psych	ip, 1st admission, 1880–1884	CR	✓	[✓]
Jablensky et al. (1992)	Colombia, Czechoslovakia, Denmark, India, Ireland, Japan, Nigeria, United Kingdom, United States, USSR	1,379	745	643	15–54	NK			ICD9: S, Par, Other P, SA, PD	1st episode contact with ‘helping agency’	IS	✓	
Kim et al. (1993)	Korea	370	199	171	NK	33.0		[✓]	DSMIIIR: S	ip, October 1991	IS	✓	
	China (Korean-Chinese)	225	137	88	NK	41.2							
	China (Chinese)	176	98	78	NK	34.9							
Tateyama et al. (1993)	Germany	150	70	80	NK	35.3			ICD9: S	ip, July–December 1984	CR	✓	
	Japan	324	158	166	NK	35.9				ip, January1983–March 1986			
Brewerton (1994)	Hawaii	50	31	19	NK	35.3	[✓]		DSMIIIR: S, S-TEEG, Aff, P-CPS	ip, 1982–1984	CR	[✓]	✓
Azhar et al. (1995)	Malaysia – Penang (Malay)	82	NK	NK	NK	NK	✓		ICD9: S	ip	IS	✓	
	Malaysia – Penang (Chinese)	84	NK	NK	NK	NK							
	Malaysia – Kota Bharu (Malay)	84	NK	NK	NK	NK							
Kanemoto et al. (1996)	Japan	33	18	15	NK	35.4			Interictal P	Archives of regional epilepsy centre	CR	✓	✓
		30	17	13	NK	37.7			Postictal P				
		25	17	8	NK	32.3			Chronic P + CPS				
Kent and Wahass (1996)	Saudi Arabia	40	NK	NK	20–65	NK			ICD10: S	ip and op	IS		✓
	United Kingdom	35	NK	NK									
Tateyama et al. (1998)	Japan	324	158	166	NK	35.9		[✓]	ICD9: S	ip, January 1983–May 1986	CR	✓	
	Austria	101	48	53	NK	35.0				ip, September 1992–December 1993			
	Germany	150	70	80	NK	35.3				ip, July–December 1984			
Appelbaum et al. (1999)	United States	1136	NK	NK	18–40	NK	✓		DSMIIIR: S, S-form, S-aff, Aff, Other P, SA, PD	ip – randomly selected	IS	✓	
Stompe et al. (1999)	Austria	126	70	56	NK	29.5		✓	DSMIIIR: S	Consecutive ip, January 1992–December 1994	IS	✓	
	Pakistan	108	73	35	NK	32.4				ip			
Kulhara et al. (2000)	North India	40	19	21	NK	32.4		✓	ICD10: S	op, 1st contact (*n* = 11 admitted)	IS	✓	[✓]
Raja, Azzoni, and Lubich (2000)	Italy	313 (cases); 271 (patients)	124	189	18–87	41.8			DSMIV: S, S-form, S-aff, Aff, Other P, Psy-ep, Other	ip – consecutive admissions to PICU 26 May 1994–10 July 1995	IS	✓	
Atallah, El-Dosoky, Coker, Nabil, and El-Islam (2001)	Egypt	5,275	NK	NK	NK	33.0	✓	✓	S, S-aff, Aff	ip, 1975–1996	CR	[✓]	✓
Getz, Fleck, and Strakowski (2001)	United States	71	42	29	18–45	32.0	✓	✓	DSMIV: P	ip – consecutive admissions	IS	✓	✓
		29	18	11		33.3							
		33	22	11		31.2							
Gutiérrez-Lobos et al. (2001)	Austria	639	239	400	15–89	48.3			ICD8: S, Aff, Org, Par, N, SA	ip, 1 January 1971–30 June 1974	CR	✓	[✓]
Kim et al. (2001)	Korea (Seoul)	143	82	61	NK	34.2		[✓]	DSMIV: S	ip, January/February 1999	IS	✓	
	China (Shanghai)	147	93	54	NK	36.5							
	Taiwan (Taipei)	140	76	64	NK	33.5							
Kim et al. (2001)	China (Shanghai)	182	119	63	NK	38.1		[✓]	DSMIV: S	ip, 1 March–30 June 1998	IS		✓
													
	Korea (Seoul)	214	125	89	NK	35.6							
													
Siddle, Haddock, Tarrier, and Faragher (2002)	England	193	135	58	18.4–64.8	NK	✓	✓	DSMIV: S, S-aff, S-form, Other P	ip, 1st admissions	IS	✓	✓
Suhail and Cochrane (2002)	England (White)	50	38	12	NK	36.5	✓		S, Par, S-aff	Ip	CR	✓	✓
	England (British-Pakistani)	53	31	22	NK	33.4							
	Pakistan	98	48	50	NK	38.4							
Suhail (2003)	Pakistan	98	48	50	NK	NK			DSMIV: S	ip, January–April 1998	IS	✓	
Smith et al. (2005)	England	20	14	6	18–65	37.1			DSMIV: S, S-aff, S-form, Aff, Other P	op and ip	IS	✓	[✓]
Miller and McCormack (2006)	United States	77	53	24	16–38	23	✓	✓	DSMIV: S, S-form, S-aff	Community hospital	IS	✓	[✓]
Rudalevičienė et al. (2008)	Lithuania	295	143	152	20–74	42.4		[✓]	ICD10: S	NK	IS	✓	
Brakoulias and Starcevic (2008)	Australia	90	49	41	18–65	37.8			S, S-aff, Aff, SA, Other P	ip, May 2006	CR	✓	
Skodlar, Dernovsek, and Kocmur (2008)	Slovenia	120	60	60	NK	NK			S	ip, 1st admission, 10 records selected for each 10-year interval, 1881–2000	CR	✓	
Gecici et al. (2010)	Turkey	373	215	158	NK	36.2	✓	[✓]	DSMIV: S	ip, January–April 2008	IS	✓	✓
Mohr et al. (2010)	Switzerland and Canada	236	150	86	NK	42.9	✓	[✓]	ICD10: S, S-aff	op: May 2003–June 2004 (Geneva); October–December 2006 (Quebec)	IS	✓	
Suhail and Ghauri (2010)	Pakistan	53	40	13	NK	35.2		✓	DSMIV: S	ip admitted July–December 2007	IS	✓	✓
de Araujo Filho et al. (2011)	Brazil	29	11	18	NK	38.5			TLE-MTS with Psy-ep	op, July 2005–July 2010	IS	✓	
		6	4	2	NK	32.6			JME with Psy-ep				
Huang et al. (2011)	Taiwan	55	22	33	NK	32.6		✓	DSMIV: S	Day-patients	IS	[✓]	[✓]
Linskey (2011)	India	50	31	19	18–72	37.7		✓	DSMIII: S, Aff	ip and op	IS	✓	
Cannon and Kramer (2012)	United States	102	48	54	NK	38.7			S, Par, Mania, Other P	ip records – randomly sampled by decade	CR	✓	
Krzystanek, Krysta, Klasik, and Krupka-Matuszczyk (2012)	Poland	400	204	196	38	NK			S	ip, 1932, 1952, 1972 and 1992	CR	[✓]	[✓]
Iyassu et al. (2014)	England	383	266	117	18–65	38.9	✓		ICD10: S, S-aff, Other P	Age 18–65 years, drawn from previous studies	IS	✓	✓
Connell et al. (2014)	South Africa	73	56	17	25–71	44	✓	✓	S	Convenience sample – participants in previous research	IS	✓	

Refer Appendix 1 for abbreviations.

**Table 2.** Empirical studies of religious delusions.

Publication	Country	*n*	Diagnosis	Prevalence of delusions		RD					Definition of RD	Information given about hallucinations
				*n*	% of total sample	*n*	% of delusional subjects	% of total sample	*n* (%) of delusional males	*n* (%) of delusional females		
Lucas et al. (1962)	England	405	S, S-aff, Par	288	71	61	21.2	15.1	23 (18)	38 (24)	No information given	
Rin et al. (1962)	Taiwan – Chinese	126	Par			12	NK	9.5	4	8	Content = ‘religion and gods’	[✓]
	Taiwan – Formosan	94				7	NK	7.4	3	4		
Weinstein (1962)	Virgin Islands	148	P			26	NK	17.6	10 (12.0)	16 (24.6)	‘Delusions and hallucinations concerning religion …’ ‘The majority of these delusions and hallucinations concerned God and Jesus’. Religion reported separately to death (within which beliefs about spirits included) and Obeah (witchcraft)	[✓]
Kiev (1963)	England	10	S			8	80	80	NK	NK	No information given	[✓]
Gordon (1965)	England	112	S, S-form, Aff, S-aff, Org, PD, N			44	NK	39.3	NK	NK	‘Religious delusions and/or religiose colouring’	[✓]
McCabe, Fowler, Cadoret, and Winokur (1972)	United States	28	Good prognosis S			13	46	46	NK	NK	No information given	✓
		25	Poor prognosis S	19	76	6	5	25	NK	NK		
El Sendiony (1976)	Egypt	110	S, Par			44	40	40	20 (36)	24 (44)	‘religious ideology’	
Ahmed (1978)	Pakistan	51	S	422	50.9	25	49	49	NK	NK	‘religious content’ *n* = 25 ‘religious and/or magic content’ *n* = 34	
Kala and Wig (1978)	India	200	ICD8: S, Par			31	15.5	15.5	8	23	‘Magic & religion’ Modified from Rin et al. (1962)	
Kala and Wig (1982)	India	200	ICD8: S, Par			41	20.5	20.5	25	16	PSE	
Ndetei and Singh (1982)	Kenya	80	All psych	62	78.4	17	27.4	21.3	NK	NK	Modified PSE. RD classified as a sub-category of grandiose delusions	
Ndetei and Vadher (1985)	England	593	All psych			20	NK	3.4	NK	NK	‘any religious symptoms regardless of whether they were delusions or just ideas’	
Cothran and Harvey (1986)	United States	41	DSMIII: S, Mania	24	58.5	13	54.2	31.7	NK	NK	‘Subjects were rated as delusional with religious content if they reported at least one delusion over the course of the SADS interview and delusional content included a report of religious state, experience, practice or belief that exceeded SADS/DSM-III criteria for legitimate subcultural experience’	
Kulhara et al. (1986)	India	112	ICD9: S	98	87.5	14	14.3	12.5	NK	NK	PSE	
Andreasen (1987)	United States	111	S	102	92	34	33.3	30.6	NK	NK	SAPS	[✓]
Renvoize and Beveridge (1989)	England	118	All psych	86	72.9	30	34.9	25.4	NK	NK	‘religious content’	[✓]
Jablensky et al. (1992)	Colombia, Czechoslovakia, Denmark, India, Ireland, Japan, Nigeria, United Kingdom, United States, USSR	1,379	ICD9: S, Par, Other P, SA, PD			NK	NK	~10.0	NK	NK	PSE Category 78	
Kim et al. (1993)	Korea	370	DSMIIIR: S	370	80.9	93	25.1	20.4	NK	NK	‘Religious, supernatural’ (Not clear if ‘possession’ counted separately)	
	China (Korean-Chinese)	225		225	77.4	0	0	0	NK	NK		
	China (Chinese)	176		176	76.2	2	1.1	0.9	NK	NK		
Tateyama et al. (1993)	Germany	150	ICD9: S	131	87	32	24.4	21.3	18	14	No information given	
	Japan	324		289	89.2	22	7.6	6.8	12	10		
Azhar et al. (1995)	Malaysia – Penang (Malay)	82	ICD9: S			9	NK	11	NK	NK	PSE	
	Malaysia – Penang (Chinese)	84				4	NK	5	NK	NK		
	Malaysia – Kota Bharu (Malay)	84				37	NK	44	NK	NK		
Kanemoto et al. (1996)	Japan	33	Interictal P			1	NK	3	NK	NK	SAPS	✓
		30	Postictal P			7	NK	23.3	NK	NK		
		25	Chronic P + CPS			0	NK	0	NK	NK		
Tateyama et al. (1998)	Japan	324	ICD9: S	290	89.50	22	6.8		12 (8.4)	10 (6.8)	Huber and Gross (1977)	
	Austria	101		92	91.10	20	19.8		9 (20)	11 (23.4)		
	Germany	150		131	87.30	32	21.3		18 (30)	14 (19.7)		
Appelbaum et al. (1999)	United States	1,136	DSMIIIR: S, S-form, S-aff, Aff, Other P, SA, PD	328	29	93	28.4	8.2	NK	NK	‘content-based typology based largely on DSM-III-R’	
Stompe et al. (1999)	Austria	126	DSMIIIR: S			27	NK	21.4	NK	NK	Huber and Gross (1977)	
	Pakistan	108				5	NK	4.6	NK	NK		
Kulhara et al. (2000)	North India	40	ICD10: S	37	92	4	10.8	10	NK	NK	PSE	[✓]
Raja, Azzoni, and Lubich (2000)	Italy	313 (cases) 271 (patients)	DSMIV: S, S-form, S-aff, Aff, Other P, Psy-ep, Other			63	NK	20.1	27	36	SAPS item on RD score >1	
Getz, Fleck, and Strakowski (2001)	United States	133	DSMIV: P			45	NK	33.8	NK	NK	SAPS item on RD score >1	✓
Gutiérrez-Lobos et al. (2001)	Austria	639	ICD8: S, Aff, Org, Par, N, SA	639	7.8	42	6.6	NK	15 (6.3)	27 (6.8)	‘religious or metaphysical’	[✓]
Kim et al. (2001)	Korea (Seoul)	143	DSMIV: S	599	92.2	67	47.1	NK	NK	NK	‘religious/supernatural’ theme ‘possession’ classified separately	
	China (Shanghai)	147				12	7.9	NK	NK	NK		
	Taiwan (Taipei)	140				57	41	NK	NK	NK		
Siddle, Haddock, Tarrier, and Faragher (2002)	England	193	DSMIV: S, S-aff, S-form, Other P			45	NK	23.3	NK	NK	PSE + Sims (1995) criteria Algorithm to establish RD	✓
Suhail and Cochrane (2002)	England (White)	50	S, Par, S-aff			7	14	14	NK	NK	PSE Category 78	✓
	England (British-Pakistani)	53				11	21	21	NK	NK		
	Pakistan	98				11	11	11	NK	NK		
Suhail (2003)	Pakistan	98	DSMIV: S			11	11	11	9	2	PSE Category 78	
Smith et al. (2005)	England	20	DSMIV: S, S-aff, S-form, Aff, Other P			11	55	55	NK	NK	Clinical Assessment in Neuropsychiatry (WHO, 1992)	[✓]
Miller and McCormack (2006)	United States	77	DSMIV: S, S-form, S-aff			36	NK	46.8	53	24	‘false fixed beliefs of a religious nature’ 36 patients identified in this study had RD ‘that significantly affected their functioning’ 2 categories of RD identified: ‘clearly’ RD and ‘delusions with religious content’	[✓]
Rudalevičienėė et al. (2008)	Lithuania	295	ICD10: S			190	NK	64.4	89 (62.2)	101 (66.4)	Semi-structured questionnaire – FPS	
Brakoulias and Starcevic (2008)	Australia	90	S, S-aff, Aff, SA, Other P	90	56	24	26.7	18.5	NK	NK	No information given	
Skodlar, Dernovsek, and Kocmur (2008)	Slovenia	120	S			38	NK	31.7	NK	NK	No information given	
Gecici et al. (2010)	Turkey	373	DSMIV: S	346	92.8	58	16.8	15.5	34 (16.8)	24 (16.7)	Huber and Gross (1977)	✓
Mohr et al. (2010)	Switzerland and Canada	236	ICD10: S, S-aff	123	52.1	38	30.9	16.1	27 (71)	8 (29)	‘delusions with religious content’	
Suhail and Ghauri (2010)	Pakistan	53	DSMIV: S			33	62.3	NK	NK	NK	PSE Category 78	✓
de Araujo Filho et al. (2011)	Brazil	29	TLE-MTS + Psy-ep			4	13.8	NA	NK	NK	No information given	
		6	JME + Psy-ep			2	33.3	NA	NK	NK		
Linskey (2011)	India	50	DSMIII: S, Aff			16	32	32	NK	NK	‘religious’ (‘magical’ & ‘spirit-possession’ classified separately)	
Cannon and Kramer (2012)	United States	102	S, Par, Mania, Other P			39	38	38	NK	NK	No information given	
Iyassu et al. (2014)	England	383	ICD10: S, S-aff, Other P	383	89.7	87	22.7	20.5	60	27	RD = Item 12 on SAPS: ‘The patient is preoccupied with false beliefs of a religious nature’	✓
Connell et al. (2014)	South Africa	73	S	60	82	42	70	57.5	NK	NK	After Drinnan and Lavender (2006) and Jones and Watson (1997)	

Refer Appendix 1 for abbreviations.

Sample size for the studies included in the total group of 55 publications ranged between 50 and 5,275 for case record studies and between 10 and 1,379 for interview studies. Less than half of the total group of publications included provided any information on the ethnicity (*n* = 22) or religious affiliation (*n* = 24) of the subject sample. A wide range of diagnostic groups was included in some studies, and in others, the sample was restricted to schizophrenia. Only three studies explicitly included psychosis related to epilepsy.

Studies were undertaken in a wide range of countries, and 11 studies explicitly included international and/or ethnic comparisons. Notably, studies appear to have been undertaken in every populated continent in the world, albeit the two countries in which many more studies have been undertaken than in any other are the United Kingdom (*n* = 12) and the United States (*n* = 10). More than half the studies (*n* = 31) included subjects from Europe and/or North America, whereas only one study included subjects from South America (Colombia).

Only five studies in the sample incorporated some kind of longitudinal analysis. Mitchell and Vierkant (1988) compared patients admitted in 1933–1939 with those admitted in 1986–1987. Skodlar, Dernovsek, and Kocmur (2008) selected case notes from each 10-year period between 1881 and 2000. Similarly, Cannon and Kramer (2011) sampled case notes by decade across the course of the 20th century. These three studies will be discussed further below. In another two studies, RD and RH were not distinguished. Atallah, El-Dosoky, Coker, Nabil, and El-Islam (2001) conducted a longitudinal analysis of case notes in a psychiatric hospital in Egypt across the period 1975–1996 and found peaks of religious symptoms in the mid-1970s to early 1980s and again in the early/mid-1990s. Krzystanek et al. (2012) studied case notes of patients admitted to a neuropsychiatric hospital in Poland in 1932, 1952, 1972 and 1992 and found religious topics identified in delusions and/or hallucinations in 50%, 46%, 49% and 42%, respectively.

Studies of RD (Table 2) have found between 1.1% and 80% of deluded subjects to report at least some religious content in their delusions. More typically, figures between 20% and 60% are reported. However, variable definitions of what counted as religious content were employed. In eight studies, no information at all was given concerning the definitions employed. Themes related to magic, death, spirit possession, witchcraft, the supernatural and so on were sometimes included and sometimes not included. Often it appears that it was taken for granted that what was ‘religious’ should be obvious to both the researcher and reader.

Skodlar et al. (2008) found that the frequency of delusions in Slovenia with religious and magical themes fluctuated during the study period 1881–2000, with low levels observed in the periods 1901–1920 and 1961–1980. Cannon and Kramer (2011) did not find variation in RD across the 20th century in the United States.

There generally seems to be a positive relationship between religiosity and RD. Cothran and Harvey (1986) and Siddle, Haddock, Tarrier, and Faragher (2002) report higher religiosity in those with RD. Getz, Fleck, and Strakowski (2001) report that religious involvement prior to admission predicted severity of RD and that Protestants are significantly more likely to report RD than Roman Catholics. Suhail and Ghauri (2010) report that more religious patients were more likely to have RD. However, Rudalevičienė, Stompe, Narbekovas, Raškauskienė, and Bunevičius (2008) concluded from their multivariate analysis that religiosity does not directly influence the religious content of delusions.

Siddle et al. (2002) reported that patients with RD had higher symptom scores, were functioning less well and were prescribed more medication. Similarly, Raja, Azzoni, and Lubich (2000) found that patients with RD started neuroleptic treatment earlier, had worse global functioning and more severe psychopathology. However, Mohr et al. (2010) reported that RD were not associated with greater clinical severity, and McCabe, Fowler, Cadoret, and Winokur (1972) found that RD did not distinguish good and poor prognosis groups of patients. Similarly, in a subsequent publication, Siddle, Haddock, Tarrier, and Faragher (2004) reported that in the subjects included in their 2002 study, after 4 weeks of treatment there was no difference in response to treatment between patients who had RD and those who did not.

Studies of RH (Table 3) provide much less quantitative information. In some studies, content of delusions and hallucinations is not distinguished and it is noted only that there is religious content to delusions and/or hallucinations. Only a few studies distinguish between religious themes appearing within the content of auditory verbal hallucinations (AVH) and ascription of a religious identity to the perceived source of the AVH. Very few studies give any significant information on hallucinations in modalities other than the auditory. As with studies of RD, definitions of what counts as ‘religious’ content of hallucinations are variable and often imprecise.

**Table 3.** Empirical studies of religious hallucinations.

Publication	Country	Study subjects			Diagnosis	Prevalence of hallucinations			Definition of RH	Information given about hallucinations	RD (% of total sample)
		Total, *n*	Male, *n*	Female, *n*		*n*	%	Type			
Rin et al. (1962)	Taiwan – Chinese	126	52	74	Par		56	AH	Content = ‘religion and gods’ – no distinction made between delusions and hallucinations	Symptom content ‘not so fluently expressed in hallucinations as in delusions’	9.5
	Taiwan – Formosan	94	45	49			52	AH			7.4
Weinstein (1962)	Virgin Islands	148	83	65	P				‘Delusions and hallucinations concerning religion …’ ‘The majority of these delusions and hallucinations concerned God and Jesus’. Religion reported separately to death (within which beliefs about spirits included) and Obeah (witchcraft)	Content of RD and RH not distinguished in this study	17.6
Kiev (1963)	England	10	NK	NK	S				NA	‘Most’ RD accompanied by ‘hallucinatory commands to preach and heal …’	80
Mott, Small, and Anderson (1965)	United States	50	14	36	S	33	66	AH	Spiritual theme = ‘seeing dead relatives, visions of spirits, etc’ Ascribed identity or sources = ‘religious personages’	*n* = 9 (18%) spirituality a major theme *n* = 8 (16%) ascribed to religious personages	NK
		50	44	6	SA (alcoholic)	38	76	AH		*n* = 12 (24%) spirituality a major theme *n* = 10 (20%) ascribed to religious personages	NK
		50	26	24	Medical	16	32	AH		*n* = 13 (26%) spirituality a major theme *n* = 6 (12%) ascribed to religious personages	NK
Gordon (1965)	England	112	61	51	S, S-form, Aff, S-aff, Org, PD, N				No information given	‘religiose content was usually associated in the schizophrenics with auditory, and often visual, hallucinations, the patients frequently seeing visions and receiving commands from God’	39.3
Scott (1967)	South Africa	100	0	100	P	85	85	AVH	No information given	44/85 = 51.8% ascribed to God	NK
McCabe, Fowler, Cadoret, and Winokur (1972)	United States	28	8	20	Good prognosis S		52	AH	No information given	More likely to have VH (*p* < .01)	46
		25	10	15	Poor prognosis S		36	AH		More likely to have ‘Special types’ of AH and haptic hallucinations (*p* < .05)	25
Littlewood and Lipsedge (1981)	England	244	NK	NK	S, Aff, Par, Other P, PD				Religious flavour defined as ‘constant preoccupation with a religious or supernatural theme, religious delusions or hallucinations, a belief in a personal religious mission or interpretation of recent events in religious or magical terms’	None	NK
Andreasen (1987)	United States	111	NK	NK	S	83	75	AH	NA	Voices commenting − 58% Voices conversing − 57%	30.6
Mitchell and Vierkant (1988)	United States	150	89	61	Delusions and hallucinations reported in files				‘perceived source of auditory hallucinations’	Perceived source of AVH include God (16), Holy Ghost/spirits (5), angels	NK
		150	89	61						Perceived source of AVH include God (3), devils/demons (9), the ‘Trinity’, Matthew (of scriptures)	
Renvoize and Beveridge (1989)	England	118	54	64	RDC: S, Aff, Other	28	24	‘mainly’ AH and VH	‘religious theme’	28.6% of patients with hallucinations had ‘a religious theme’	25.4
Brewerton (1994)	Hawaii	50	31	19	DSMIIIR: S, S with temporal lobe EEG abnormalities, Aff, P secondary to CPS				‘clearly noted religious themes as part of the delusions and/or hallucinations’ Religion defined according to Webster’s Dictionary: ‘belief in a divine or superhuman power or powers to be obeyed and worshipped’ or ‘any specific system of belief, worship, conduct, etc, often involving a code of ethics and a philosophy’	AVH were typically of God (*n* = 14), devil/demons (*n* = 12) or spirits/saints (*n* = 4) RD and/or RH = 74% of total	NK
Kanemoto et al. (1996)	Japan	33	18	15	Interictal P				No information given	*n* = 9 voices commenting *n* = 12 other AH *n* = 6 somatic/tactile hallucinations	3
		30	17	13	Postictal P					*n* = 1 voices commenting *n* = 3 other AH *n* = 5 somatic/tactile hallucinations	23.3
		25	17	8	Chronic P + CPS					*n* = 8 voices commenting *n* = 11 other AH *n* = 3 somatic/tactile hallucinations	0
Kent and Wahass (1996)	Saudi Arabia	40	NK	NK	ICD10: S				Religious themes = ‘relationship between the patient and his god, eg instructions to read a holy book, chastisement after death, or mention of paradise’ Superstitious content = ‘mention of demons, magic and spirits’	Second-person voices 53% religious Third-person voices 33% religious	NK
	United Kingdom	35	NK	NK						Second-person voices 11% religious Third-person voices 6% religious	
Kulhara et al. (2000)	North India	40	19	21	ICD10: S	28	70	Any	No information given	70% hallucinated No information on content	10
Atallah, El-Dosoky, Coker, Nabil, and El-Islam (2001)	Egypt	5,275	NK	NK	S, S-aff, Aff				‘Religious symptoms’ defined as all symptoms with religious content, including ‘everything from increased praying or reading religious books, increased religiosity, spending all one’s time in the church or mosque, to believing oneself to be (or be married to) a religious figure, on a religious mission to save the world, and so on. In addition, supernatural beliefs such as black magic (A’mal), demon possession, or the evil eye were included’	*n* = 135/632 patients with religious symptoms (21.3%) had auditory RHs, 105 (16.62%) had visual RHs and 12 (1.9%) had tactile RHs Hallucinations equally common among patients with and without religious symptoms	NK
Getz, Fleck, and Strakowski (2001)	United States	71	42	29	DSMIV: P				No information given	SAPS Hallucination Score = 3.4SAPS Hallucination Score = 3.6SAPS Hallucination Score = 3.2	43
		29	18	11							20.7
		33	22	11							24.2
Gutiérrez-Lobos et al. (2001)	Austria	639	239	400	ICD8: S, Aff, Org, Par, Other P, N, SA				NA	Mean age for 1st hearing voices 44.4 years	NK
Kim et al. (2001)	China (Shanghai)	182	119	63	DSMIV: S	101	56	Any	‘Religious/supernatural themes’	12 (11.9%) AH with supernatural/religious identity 12 (12.2%) AH with religious/supernatural theme	NK
						100	55	AH			
	Korea (Seoul)	214	125	89		130	61	Any		37 (28.5%) AH with supernatural/religious identity 41 (36%) AH with religious/supernatural theme	
						128	60	AH			
Siddle, Haddock, Tarrier, and Faragher (2002)	England	193	135	58	DSMIV: S, S-aff, S-form, Other P		<50	AH	No information given	Over 50% of the sample reported no AH RD most commonly secondary to hallucination RD more likely to indicate more certainty in an external cause for their voices than an internal cause	23.3
Suhail and Cochrane (2002)	England (White)	50	38	12	S, Par, S-aff	44	88	AVH	Voices identified as God	Voice of God: 5 (10%)	14
						13	26	VH			
	England (British-Pakistani)	53	31	22		38	72	AVH		Voice of God: 5 (9%)	21
						13	24	VH			
	Pakistan	98	48	50		51	52	AVH		Voice of God: 6 (6%)	11
						6	6	VH			
Smith et al. (2005)	England	20	14	6	DSMIV: S, S-aff, S-form, Aff, Other P	7	35	AH	Clinical Assessment in Neuropsychiatry (WHO, 1992)	*n* = 7 had AH	55
Gecici et al. (2010)	Turkey	373	215	158	DSMIV: S	236	63	AH	AVH classified according to source (God/Prophet/Devil) VH classified according to object seen (Prophet, Devil, God, saint)	*n* = 15 voices from God, *n* = 10 voices from the Prophet, *n* = 9 voices from the Devil (VH n=9, n=11, n=10 respectively)	15.5
Suhail and Ghauri (2010)	Pakistan	53	40	13	DSMIV: S		74	AVH	No information given	More/less religious patients did not differ on AVH (65% vs 76%)	NK
							59	VH		VH of spirits/ghosts/jinee/holy – *n* = 3 (12%) in less religious group and *n* = 14 (50%) in more religious group	
							55	Olfactory			
de Araujo Filho et al. (2011)	Brazil	29	11	18	TLE-MTS with Psy-ep	29	100	Any	NA	None	NA
		6	4	2	JME with Psy-ep	6	100	Any			
Huang et al. (2011)	Taiwan	55	22	33	DSMIV: S				PANSS ‘The term “religion” was defined as belief in divine or superhuman power or powers to be obeyed and worshipped; or any specific system of belief, worship, conduct, etc, often involving a code of ethics and a philosophy’	7 (12.7%) of total sample had ‘psychopathology with religious content’ (RD, RH or ritual behaviour) RD/RH related to higher religiosity RD/RH associated with lower satisfaction with psychiatric therapy and received more magico-religious healing	NK
Krzystanek, Krysta, Klasik, and Krupka-Matuszczyk (2012)	Poland	400	204	196	S				‘symptoms with religious content’ Thematic groups = the Holy Trinity, the Virgin Mary, the Bible, saints, religious imagery, church and names of deities	None specifically	NK
Iyassu et al. (2014)	England	383	266	117	ICD10: S, S-aff, Other P	248	65	Any	No information given	RD group scored more highly on hallucinations	20.5

Refer Appendix 1 for abbreviations.

Mott, Small, and Anderson (1965) observed spiritual themes in 18%−26% of AVH. Renvoize and Beveridge (1989) found that 28.6% of patients with hallucinations (which were ‘mainly auditory and visual’) had a religious theme. Atallah et al. (2001) found that only 135 (21.3%) out of 632 patients with religious symptoms had auditory RH. In the same study, 105 (16.2%) had visual RH and 12 (1.9%) had tactile RH. Kim et al. (2001) found religious/supernatural themes in 12.2% of the auditory hallucinations of their Chinese subjects and in 36% of their Korean subjects. Kent and Wahass (1996) found that religious themes were less common in hallucinations experienced by subjects in the United Kingdom than in Saudi Arabia and also less common in third-person voices than in second-person voices. Mitchell and Vierkant (1988) found that command hallucinations more often included religious content in the 1930s than in the 1980s.

Mott et al. (1965) found that 16%−20% of AVH were ascribed to religious personages. Scott (1967) found that 51.8% of AVH in a study in South Africa were ascribed to God. Kim et al. (2001) found that a religious/supernatural identity was ascribed to the source of the voices in 11.9% of their Chinese subjects and 28.5% of their Korean subjects. Suhail and Cochrane (2002) found that 10% (*n* = 5) of their White English subjects and 9% (*n* = 5) of their British-Pakistani subjects, but only 6% (*n* = 6) of their Pakistani subjects living in Pakistan, reported hearing voices which they identified as God. In a sample of 373 patients with schizophrenia in Turkey, Gecici et al. (2010) identified only 15 subjects who heard voices that they believed to be from God, 10 who heard the voice of the prophet Mohammed and 9 who heard voices from the devil.

The relationship between RD and RH seems to have received surprisingly little attention. In a small and early study of West Indian immigrants in London, Kiev (1963) reported that ‘most’ RD were accompanied by ‘hallucinatory commands to preach and heal …’ In a similar but larger early study, Gordon reported that

The religiose content was usually associated in the schizophrenics with auditory, and often visual, hallucinations, the patients frequently seeing visions and receiving commands from God.

Suhail and Ghauri (2010) report that more religious patients are both more likely to experience RD and to hear voices of ‘paranormal agents’. Siddle et al. (2002) report that RD occur most commonly secondary to RH. Iyassu et al. (2014) reported that 75.9% of those with RD and 61.7% of those with other delusions had ‘anomalous experiences’ (by which they meant hallucinatory experiences in any modality).

## Discussion

Religious content of delusions and hallucinations would appear to be relatively common, and yet there is a lack of agreed definition as to where the boundaries of what is truly ‘religious’ lie. Even where standardised instruments such as the Present State Examination (PSE) or Scale for the Assessment of Positive Symptoms (SAPS) have been used, much is left to the discretion of the researcher. The lack of definition provides further cause for concern where, in some studies, little or no attention appears to have been paid to the religious affiliation or context of the research subjects. In the case of RH, only a few studies have distinguished between content and identity or source of AVH. All of this raises the important question of what properly constitutes ‘religious’ content of delusions and/or hallucinations.

To take a narrower view of things, it might be argued that religious content should be understood to reflect or refer to traditional religious beliefs, persons or stories. Thus, references to ‘sin’ (as opposed to more general concerns of morality), divinity, resurrection or reincarnation, and witchcraft would all appear to qualify as religious, as would references to figures such as Buddha, Jesus or Mohammed. However, much traditional religious belief has now become detached from its original context and is upheld by those who follow newer spiritual paths which they may determine as ‘spiritual but not religious’. For example, spirit possession is a feature of various religious traditions, including the major monotheistic faiths, but interaction with spirits of various kinds is also seen in the so-called New Age spiritualities. References to the supernatural, superstition, magic and voices of (or delusions concerning) the dead are similarly ambiguous.

To broaden the category of interest to ‘spiritual’ (rather than religious) would be in danger of making the boundaries even more blurred. However, definitions of spirituality generally encompass relatively few subsidiary concepts (Cook, 2004), and these might prove to be more helpful categories for future research. For example, delusions might be classified according to whether they refer to immanent or transcendent relationships. (Immanent relationships refer to those with people and things in the natural order and transcendent relationships to those with a non-material, spiritual or divine order understood as being above and beyond the natural. For further discussion, see Cook, 2013.) As Koenig, King, and Carson (2012) have pointed out, definitions of religion and spirituality commonly emphasise broadly transcendent over immanent concerns (although see also Cook (2013)). Similarly, content might be classified according to reference to matters of meaning or purpose in life, concepts of life-force or soul, ultimate concerns and other deeply held values, all of which may reflect either religious concerns or spiritual-but-not-religious concerns, or perhaps both of these or neither of these.

An important difference between delusions and hallucinations is that delusional thought (with the important exception of thought insertion) is generally owned as ego-syntonic. Hallucinations are identified as originating from external agency, and so the source or identity of that agency becomes a separate, albeit related, concern to the matter of the content of the hallucination. Few studies to date have clearly or carefully addressed this important distinction, and the identity of AVH has often not been clarified. Thus, for example, the author once encountered a patient who reported what appeared to be an olfactory hallucination of the smell of rotting meat, which in itself is not a religious theme. However, taken in the whole context of the clinical history, and in particular of a delusional belief that she was demon-possessed, this hallucination had clear religious significance and was attributed by the patient to the activity of evil spirits.

It is therefore not immediately apparent that there is a simple answer as to how RD/RH should be defined, but it is clear that better characterisation and description of terms within future research will be important. It would also appear likely that the prevalence of RD and RH may have been underestimated in at least some studies.

Notwithstanding these concerns, the frequency of occurrence of RD and RH does clearly appear to vary widely with time and place. In most cases, as in the comparisons between Saudi Arabia and the United Kingdom (Kent & Wahass, 1996) or Korea and China (Kim et al., 2001), it would appear likely that this reflects an influence of culture and environment on the individual. The work of Suhail and Cochrane (2002) suggests that the culture in which one lives may be more important than country of origin in determining whether or not the source of RH is identified as being from God.

However, within any given environment, and notwithstanding the findings of Rudalevičienė et al. (2008), it might also be expected that personal religiosity would also play a part. Thus, personal beliefs that precede any illness, disorder or disturbance would be expected to contribute to shaping the content of psychopathology.

Some support for the impact of personal religiosity may be found in other published research. In normal volunteers without mental illness who are subjected to a primed word-detection task, subjects high in religiosity are more likely to report false perception with religious content than are those low in religiosity (Reed & Clarke, 2014). In a study of 1,006 subjects with schizophrenia, undertaken across six different countries, 15.5% of Roman Catholics, but only 3.8% of Muslim patients, reported delusions of guilt, suggesting that religious confession may influence delusional content independently of culture (Stompe et al., 2006). On the other hand, qualitative research involving subjects with RD suggests that it is clearly possible to be influenced by religious beliefs without considering oneself to be religious (Drinnan & Lavender, 2006, p. 326).

It must also be the case that the content of primary psychopathology itself plays an important part in shaping the content of other psychopathology. Very few studies appear to have addressed this, but where they have given the matter attention it appears to be agreed that the content of RH is often the primary basis for forming secondary RD. In principle, there would seem to be no reason to suppose that the reverse relationship might not also occur – that is, that the religious content of delusions is determinative of the religious content of hallucinations. More research on this would appear to be needed.

Notwithstanding reports in the German literature (Stompe et al., 2003) that RD are less common than formerly, it is not entirely clear that they are in continued or consistent decline in the 20th and 21st century studies included in the present review. The retrospective case note studies included in the present review showed a fluctuating rather than inexorably declining prevalence of RD. Furthermore, if we observe in Table 2 the proportion of delusional subjects reporting RD in studies undertaken in any one country (e.g. the United States or the United Kingdom) over the last 50 years or so, we do not gain a clear picture of steady decline but rather of fluctuation.

The research findings considered here suggest that religious content of delusions and hallucinations, and the perceived source of RH, may not always be identified in clinical practice. More careful enquiry into the relationship between faith (or spirituality) and psychopathology might elicit a fuller understanding of the patient’s beliefs and experiences. This may be important in helping patients to feel more fully understood and, if handled sensitively, in building trust. In some cases, it may also have diagnostic significance.

Given that we now know that voices are heard in religious contexts which are not necessarily associated with major mental illness and that some voice hearers appear to derive benefit from dialogue with their voices (Luhrmann, 2012a), the question arises as to whether or not engagement of dialogue with RH might be helpful in the course of treatment.

## Conclusion

RD and RH are commonly encountered in major mental illness, albeit prevalence varies according to time, place and personal religiosity. Comparisons between studies, and accurate estimates of prevalence, are hampered by lack of clear working definitions of exactly what constitutes a ‘religious’ delusion or hallucination and also by failure to obtain data on religious affiliation of research subjects. There is need for more critical attention to these issues in research design, and it is proposed here that a focus on transcendent concerns may well prove fruitful for future research, especially within multi-ethnic groups, and in other contexts where there is a plurality of religious belief and affiliation. Study of RH has especially been neglected, and more attention needs to be paid in future research to hallucinatory experiences in all modalities, rather than focusing almost exclusively on AVH, to distinguish between the content of the hallucination and its believed source or identity and to establish whether the RD or RH constitute the primary source of religious themes.

## Appendix 1

### Abbreviations

Aff affective disorder
All psych all psychiatric diagnoses
CPS complex partial seizures
JME juvenile myoclonic epilepsy
MTS mesial temporal sclerosis
N neurosis
Org organic psychosis
Other P other Psychosis
Par paranoid psychosis
P psychosis (any/all – unless otherwise specified)
P-CPS psychosis secondary to complex partial seizures
PD personality disorder
Psy-ep psychosis of epilepsy
S schizophrenia
S-aff schizoaffective disorder
S-form schizophreniform disorder
SA substance abuse
S-TEEG schizophrenia with temporal lobe EEG abnormalities
TLE temporal lobe epilepsy
ip in-patients
op out-patients
CR case record study
IS interview study
AH auditory hallucinations
AVH auditory verbal hallucinations
NA not applicable
NK not known
PICU psychiatric intensive care unit
RD religious delusions
RH religious hallucinations
VH visual hallucinations
FPS Fragebogen fur Psychotische Symptome
PANSS Positive and Negative Symptom Scale
PSE Present State Examination
SADS Schedule for Affective Disorders and Schizophrenia
SAPS Scale for the Assessment of Positive Symptoms
DSM *Diagnostic and Statistical Manual of the American Psychiatric Association*
ICD International Classification of Diseases
RDC Research Diagnostic Criteria
✓ Information provided in the publication
[✓] Some information provided in the publication
